# Open Source High Content Analysis Utilizing Automated Fluorescence Lifetime Imaging Microscopy

**DOI:** 10.3791/55119

**Published:** 2017-01-18

**Authors:** Frederik Görlitz, Douglas J. Kelly, Sean C. Warren, Dominic Alibhai, Lucien West, Sunil Kumar, Yuriy Alexandrov, Ian Munro, Edwin Garcia, James McGinty, Clifford Talbot, Remigiusz A. Serwa, Emmanuelle Thinon, Vincenzo da Paola, Edward J. Murray, Frank Stuhmeier, Mark A. A. Neil, Edward W. Tate, Christopher Dunsby, Paul M. W. French

**Affiliations:** ^1^Photonics Group, Department of Physics, Imperial College London; ^2^Institute for Chemical Biology, Department of Chemistry, Imperial College London; ^3^MRC Clinical Sciences Centre, Hammersmith Hospital; ^4^Chemical Biology Section, Department of Chemistry, Imperial College London; ^5^Retroscreen Virology Ltd; ^6^Pfizer Global Research and Development, Pfizer Limited, Sandwich, Kent, UK; ^7^Centre for Histopathology, Imperial College London

**Keywords:** Biophysics, Issue 119, fluorescence microscopy, FLIM, HCA, open source, FRET, automated microscopy, drug discovery

## Abstract

We present an open source high content analysis instrument utilizing automated fluorescence lifetime imaging (FLIM) for assaying protein interactions using Förster resonance energy transfer (FRET) based readouts of fixed or live cells in multiwell plates. This provides a means to screen for cell signaling processes read out using intramolecular FRET biosensors or intermolecular FRET of protein interactions such as oligomerization or heterodimerization, which can be used to identify binding partners. We describe here the functionality of this automated multiwell plate FLIM instrumentation and present exemplar data from our studies of HIV Gag protein oligomerization and a time course of a FRET biosensor in live cells. A detailed description of the practical implementation is then provided with reference to a list of hardware components and a description of the open source data acquisition software written in µManager. The application of *FLIMfit*, an open source MATLAB-based client for the OMERO platform, to analyze arrays of multiwell plate FLIM data is also presented. The protocols for imaging fixed and live cells are outlined and a demonstration of an automated multiwell plate FLIM experiment using cells expressing fluorescent protein-based FRET constructs is presented. This is complemented by a walk-through of the data analysis for this specific FLIM FRET data set.

**Figure:**
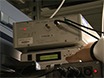


## Introduction

The increasing use of automated high content analysis (HCA) in drug discovery^1^ to assay compound libraries is complemented by the trend in life science basic research towards automated imaging experiments for systematic studies, *e.g.*, for phenotypic screens of siRNA libraries. Automated HCA is also becoming increasingly important for smaller scale biological studies that have typically been implemented with manual experiments with tens of dishes of cells imaged with conventional microscopes. The statistical power conferred by the ability to assay and analyze hundreds to thousands of fields of view enables averaging of experimental noise (including "biological noise") and the automation of image data acquisition and analysis of large sample arrays can help eliminate operator bias and often may be utilized to detect the occurrence of systematic errors, such as a change in the IRF profile during an acquisition. Fluorescence based assays are widely implemented in HCA, with most commercially available HCA instrumentation featuring fluorescence intensity imaging in one or more spectral channels to map the relative distribution and co-localization of labeled proteins. More sophisticated fluorescence imaging modalities, such as fluorescence lifetime imaging (FLIM), polarization anisotropy imaging and time-resolved fluorescence anisotropy imaging, are not widely exploited in commercial HCA instruments, although they offer powerful quantitative readouts, *e.g.,* of protein interactions. Here we present an open source approach to implementing FLIM in an automated microscope for HCA.

Fluorescence lifetime provides an inherently ratiometric spectroscopic readout that can be used to distinguish different molecular species or to probe the local molecular environment of a fluorophore and is insensitive to fluorophore concentration, to excitation/detection efficiencies, and to the impact of scattering and sample absorption^2^. Furthermore, since fluorescence lifetime measurements can be made in a single spectral channel, they are directly comparable between different instruments and samples without calibration. They can be applied to endogenous fluorophores, including some cellular metabolites, lipids and extracellular matrix components, to provide intrinsic readouts of biological processes and pathologies. Thus label-free FLIM can map metabolic changes in cells^3,4^ and has been applied to monitor stem cell differentiation^5,6^ and the growth of collagen scaffolds^7^. We recently demonstrated that FLIM HCA can be used for automated label-free assays of cellular metabolism utilizing autofluorescence^8^. More commonly, however, fluorescence lifetime measurements and FLIM are applied to exogenous fluorophores that label specific biomolecules, often implemented using dyes that stain cellular compartments, using dyes coupled to antibodies that target specific proteins in fixed cells, or using genetically expressed fluorophores coupled to specific proteins. Fluorescence lifetime can be used to provide robust quantitative readouts of fluorescent probes sensing their physical or chemical environment. For examples, dyes have been deployed to sense the local lipid phase of cell membranes^9^ or cell viscosity^10^ and genetically expressed fluorescent protein-based biosensors have been developed to report concentrations of cell signaling molecules including as IP3^11^, PIP2^12^ and calpain^13^ or ions such as calcium^14,15^, potassium^16^ and chloride^17^.

Many such biosensors utilize Förster Resonance Energy Transfer (FRET)^18,19^ to provide readouts of changes in the biosensor conformation. FRET between "donor" and "acceptor" fluorophores occurs via a short range direct dipole-dipole interaction for which fluorophores are typically separated by less than ~10 nm. Thus the detection of FRET indicates the close proximity of appropriately labeled proteins and hence provides a means to read out protein-protein interactions^20^, such as binding or oligomerization — either as end points in fixed cells or as dynamic events in time course measurements of live cells. By labeling an appropriate molecular construct with both donor and acceptor fluorophores, it is also possible to read out conformational changes — or cleavage — of the construct via the change in FRET and this is the basis of many biosensors^21^. Measurements of the FRET efficiency can provide information about the donor-acceptor distance and the population fraction of donor fluorophores undergoing FRET. Thus, FRET-based imaging techniques can be used to map and quantitate molecular interactions in space and time, *e.g.,* to elucidate cell signaling processes^22^. Automating such imaging techniques could provide high throughput assays based on readouts of signaling pathways or networks.

In HCA, the FRET readout most commonly implemented is spectral ratiometric imaging, where changes in the ratio of acceptor to donor intensity are mapped. While this approach is readily implemented — and is available in many commercially available multiwell plate readers — quantitative spectrally resolved FRET measurements require calibration of the spectral response of the system^23^. This includes spectral cross-talk arising from spectral bleed-through and direct excitation of the acceptor as well as the transmission characteristics of the instrument and the sample (inner filter effect). In principle, this entails measurement of additional "control" samples that are labeled with either donor-only or acceptor-only.

From such spectral ratiometric FRET measurements, an *effective FRET efficiency* (the product of the actual FRET efficiency and fraction of FRETing donor/acceptor molecules) can be obtained along with the relative proportion of donor and acceptor molecules^24^. Spectral ratiometric FRET is often used with unimolecular FRET biosensors, where the donor/acceptor stoichiometry can be assumed to be known. To determine the population fractions of the FRETing donor and acceptor, *e.g.*, to obtain a dose response curve, knowledge of the actual FRET efficiency is required. This can be obtained by measuring a positive FRET control sample with known properties or by using a different method to measure the FRET efficiency, such as acceptor photobleaching or FLIM.

Using fluorescence lifetime readouts, FRET can be detected and quantified by measurements of the donor emission alone — removing the need for spectral calibration and further control samples. Thus, FLIM FRET readouts can be compared between instruments and between different samples — allowing data from endoscopy or tomography of live disease models^25^ to be benchmarked against measurements of cell-based assays. By fitting donor fluorescence decay profiles to an appropriate multi-exponential decay model corresponding to FRETing and non-FRETing donor populations, FRET efficiencies and population fractions can, in principle, be obtained directly without further measurements. These significant advantages, however, come at the cost of FLIM requiring more detected photons per pixel than spectrally resolved intensity imaging — typically hundreds of photons are required to achieve an accuracy in lifetime determination of 10% when fitting to a single exponential decay model and when fitting fluorescence decay profiles to complex (multiexponential decay) models, the number of detected photons required increases to thousands. This has conventionally led to long acquisition times (tens to hundreds of seconds per field of view) for FLIM, particularly when using time correlated single photon counting (TCSPC) implemented with sequential pixel acquisition in laser scanning microscopes. Attempts to increase the imaging speed by using higher excitation intensities can lead to significant photobleaching and/or phototoxicity. Together with a lack of appropriate commercially available instrumentation and software tools, this has hindered FLIM from being utilized in HCA for drug discovery or systems biology, where hundreds to thousands of fields of view are to be imaged. Laser scanning TCSPC FLIM has been implemented in an automated multiwell plate microscope^26^ for secondary measurements following identification of "hits" by steady-state polarization-resolved fluorescence anisotropy imaging^27^, which can reach similar imaging speeds to spectral ratiometric imaging^28^ with which it shares many advantages and disadvantages. Fluorescence anisotropy imaging exploits the decrease in fluorescence anisotropy of the acceptor in the presence of FRET and is also sensitive to spectral cross-talk (*e.g.,* direct excitation of the acceptor). It requires calibration to account for polarization cross talk and does not provide a direct measurement of FRET efficiency.

To realize the advantages of FLIM for HCA, we have worked to address the issues of speed of image acquisition and wider access to the technology. Here, we provide a complete list of open source software and hardware components that can be utilized to implement the automated rapid FLIM multiwell plate reader that is described below, together with exemplar FRET based-assays. In our approach^29^, FLIM is realized using wide-field time-gated imaging using a gated optical image intensifier (GOI), which is illustrated in **Figure 1** that can provide faster imaging rates and lower photobleaching than single-beam scanning TCSPC due to the parallel pixel interrogation. Our *openFLIM-HCA* instrument can provide unsupervised FLIM, requiring only a few seconds to acquire FLIM data detecting a few 100 photons per pixel (depending on the brightness of the sample). Combined with the time required to move the sample from the previous field of view, to adjust the acquisition settings and to maintain the sample in focus, this typically results in multiwell plate acquisition times of ~10 s per field of view^25,28^. Optical sectioning can be implemented using a quasi-wide-field Nipkow ("spinning disc") scanner^30^.

For FLIM data analysis, we have developed an open source software tool called *FLIMfit*^31^ that is able to rapidly analyze multiwell plate FLIM datasets on conventional PC workstations and is a plug-in for OMERO^32^. *FLIMfit* provides global analysis capabilities (discussed in Section 1.2) that enable FLIM data to be fitted to complex models with only a few 100 photons per pixel under the assumption that the fluorescence lifetime components are invariant across an image or region of interest (ROI), therefore reducing the number of detected photons required, the light exposure to the sample and image acquisition time. Running *FLIMfit* on a standard desktop PC, requires only 10s of seconds of computational time to analyze data sets comprising hundreds of FOVs. Thus both FLIM data acquisition and analysis can be undertaken on timescales that are practical for HCA.

### *openFLIM-HCA* hardware

Our implementation of FLIM HCA comprises six key components: (i) a fluorescence microscope frame with optical autofocus; (ii) a motorized (x-y-z) microscope stage; (iii) an excitation laser source; (iv) a wide-field time-gated FLIM system with gated optical intensifier (GOI), delay generator and camera; (v) a computer for data acquisition and analysis and (vi) a Nipkow disc scanner unit for optically sectioned imaging to suppress out of focus light. This can be important when imaging weak signals, *e.g.*, from FRET biosensors at the cell plasma membrane, that could be swamped by contributions from cytoplasmic fluorescence or background from coverslips or culture medium. Time-gated FLIM data is acquired by illuminating the sample with the ultrashort pulsed excitation radiation, imaging the fluorescence emission to the photocathode of the GOI and acquiring a series of CCD images of the phosphor of the GOI for a range of settings of time delay of the optical intensifier gate after the arrival of the excitation pulses. As the time gate delay following excitation is increased, the fluorescence signal is seen to decrease and the series of time-gated fluorescence intensity images acquired on the CCD form the data set required to analyze the decay profiles of all the fluorophores in the field of view. The gating of the GOI is synchronized to the excitation pulses and, for the series of CCD acquisitions, the delay of the time gate relative to the excitation pulses is set to a series of increasing values over the desired range. This synchronization is achieved using the delay generator that comprises a "fast delay box" (providing delays up to 20 ns in 1 ps steps) and a "coarse delay box" that provides delays with a minimum step of 25 ps.

For FLIM, excitation can be provided by any ultrafast laser. For convenience, we typically employ a fiber-laser-pumped supercontinuum source that provides picosecond pulses tunable across the visible spectrum from which the appropriate excitation radiation can be selected for any fluorophore using a motorized filter wheel with different band pass filters. Typically, we use an average power of ~100-200 µW at the sample with pulses at 40 or 60 MHz repetition rate. Such excitation powers could also be provided by ultrafast laser sources based on frequency doubling of mode-locked titanium doped sapphire lasers. Note that an excitation pulse duration below ~100 ps is sufficient for most experiments. A second motorized filter wheel equipped with neutral density filters is used to regulate the excitation intensity. For safety and convenience, the excitation radiation may be delivered via a single mode optical fiber. The configurations for wide-field FLIM and optically sectioned FLIM are presented in **Figures 2 **and** 3** respectively. For more details of the precise components used, please visit the openFLIM-HCA wiki^33^. A copy of the current parts list is given in the Supplementary Material.

For wide-field FLIM, the excitation radiation is required to illuminate the whole field of view as uniformly as possible. For this we direct the excitation laser beam to a holographic "top-hat" diffuser that is rotated at 10-50 Hz to time-average the laser speckle, with the plane of the diffuser being imaged to the back focal plane of the objective microscope lens. The fluorescence image from the output port of the microscope is relayed onto the photocathode of the GOI and the phosphor of the GOI (active area diagonal 18 mm) is imaged onto the sensor of a cooled scientific CCD (chip size 10.2 mm x 8.3 mm) camera using a pair of camera lenses providing a magnification of 0.7. The system is controlled by a standard PC that is interfaced to the instrumentation using RS232 protocols. In addition, an Arduino microprocessor is used to control a shutter in the excitation light path that is closed except when the CCD camera is acquiring FLIM data and to record the signal from a photodiode that is used to measure the average power from the spectrally filtered supercontinuum excitation source. For optically sectioned FLIM, the excitation laser radiation is coupled into a single-mode polarization-maintaining optical fiber that connects directly to the Nipkow disc scanner unit, as shown in **Figure 3**. The output fluorescence image from the Nipkow scanner unit is relayed onto the photocathode of the GOI and the rest of the system is the same as for wide-field FLIM.

### *openFLIM-HCA* software

The data acquisition software is written in *µManager*^34^. It provides full HCA data acquisition functionality including options to change the sequence in which the wells of the plate are imaged, to acquire time courses for any FOV, to automatically maintain focus during measurements and to control the excitation and emission filter wheels and the dichroic changer for automated multicolor imaging. It is also possible to run a "prescan" acquisition of fluorescence intensity in order to automatically identify and record the positions of suitable FOVs for a subsequent FLIM acquisition according to criteria, *e.g.*, based on intensity. FLIM HCA data can be saved as a series of TIFF files, as a series of OME-TIFF files (*i.e.*, one per FOV) or as a single OME-TIFF file incorporating all the data from a multiwell plate. More information and a detailed description of the µManager software can be found on the openFLIM-HCA wiki^33^.

The data analysis software, *FLIMfit^31^*, an open source MATLAB-based client for the OMERO image data management platform^32^, is able to read FLIM data from an OMERO server or from computer disk, as indicated in **Figure 4**. It has been designed to analyze data from TCSPC or wide-field time-gated FLIM instruments and provides many capabilities to fit data from openFLIM-HCA including fitting to monoexponential decay profiles, and to double exponential and higher complexity decay profiles, including models such as polarization-resolved fluorescence decay profiles. It can also take account of fixed and time-varying background signals and can utilize a measured spatio-temporal instrument response function (IRF) or can fit using an idealized IRF that may be time-shifted on a pixel-wise basis by an amount determined from the full experimental IRF measurement. A global time difference (*t_0_*) between the IRF and a fluorescent sample can be determined from a measurement of a fluorescence standard. Spatial variations in background signals and IRF can also be taken into account. *FLIMfit* is able to fit FLIM data on a pixel-wise basis or using "*global binning*" or "*global fitting*" to facilitate fitting of FLIM data with modest (hundreds) photon numbers to complex decay models.

*Global binning* entails aggregating all the detected photons from the pixels within a user-defined ROI at each time point to provide sufficient photon numbers to enable fitting to complex decay models. The ROI can be the entire field of view (FOV), it can be manually defined by the user, or it can be determined using image segmentation tools. The ROI can also be defined using masks imported into *FLIMfit* from other image segmentation software. For FRET applications, it is common to use global binning to fit to a double exponential decay model to determine the fluorescence lifetime components corresponding to FRETing and non-FRETing donor fluorophores that are assumed to be spatially invariant across the ROI and then to refit on a pixel-wise basis with these lifetime component vales fixed in order to obtain the FRETing donor population fraction in each pixel.

*Global fitting* entails simultaneously fitting the lifetime components and pixel-wise FRETing donor population fractions across a whole FOV- or across a FLIM data set that could comprise multiple FOVs, *e.g.*, from a multiwell plate experiment or a time series of fluorescence lifetime images. Global fitting is able to exploit the spatial variation of population fractions across the image that is lost in the global binning approach to provide stronger constraints on the component lifetime estimation — and therefore more reliable results — albeit at the cost of significantly more computation^35^. *FLIMfit* can rapidly analyze multiwell plate FLIM datasets with global fitting on conventional PC workstations with, for example, a multiwell time-gated FLIM data set, acquired with five time gates for each of 394 FOV each comprising 672 x 512 pixels, requiring only 32 seconds and 2 GB of memory to fit to a double exponential decay model^31^. This has been achieved by utilizing a separable nonlinear least square fitting algorithm implemented using multithreaded parallel algorithms to enable effective scaling on multicore processors and the *FLIMfit* code has been optimized to minimize memory usage. **Figure 5** shows a snapshot of the graphical user interface of *FLIMfit* and more details can be found at the web page given in reference 36. Further information on global fitting and global binning can be found in reference 31.

The following protocol for FLIM HCA using our *openFLIM-HCA* instrumentation and software can be adapted for a wide range of cell imaging experiments with FLIM readouts. In general, multiwell plate FRET experiments should be designed to include replicate wells of positive and negative biological controls in addition to the conditions being studied. Here, we describe the specific implementation to perform optically sectioned FLIM (see **Figure 3**) of Cos cells expressing FRET constructs consisting of the mTurquoise fluorescent protein and Venus fluorescent protein joined by a short peptide linker. Here the mTq32V, mTq17V and mTq5V FRET constructs (discussed in Representative Results section) provide low, medium and high FRET efficiencies together with the non-FRETing mTq5A construct. These constructs and the other materials required to follow this protocol are listed in the Materials List.

## Protocol

### 1. Preparation of Multiwell Plate Array

1. Prepare a solution of 75 μM Coumarin 6 in ethanol (τ ~ 2.43 ns) for reference measurements to enable determination of *t_0_*.
2. Seed 150,000 Cos cells into four wells of a 6 well plate and allow to attach overnight in culture medium (see Materials List).
3. Transfect one well each with mTq5A, mTq5V, mTq17V and mTq32V using a commercial transfection reagent according to the manufacturer's instructions and culture for 24 h.
4. Detach cells using a commercial trypsin/EDTA solution according to manufacturer's protocol.
5. Pipette 5,000 Cos cells suspended in Hank's Balanced Salt Solution (HBSS) expressing mTq5A into each of wells A1-G3 of a #1.5 thickness glass-bottomed 96 multiwell plate that has previously been treated with poly-L-lysine. Repeat for mTq5V expressing cells into wells A4-G6, mTq17V into wells A5-G9 and mTq32V into wells A10-G12.
6. Leave well H1 empty (to provide measurement of any static background from the multiwell plate).
7. Pipette 200 µL of HBSS only into well H2 (to provide measurement of any background signal from the cell culture medium).
8. Pipette 5,000 non-transfected cells in HBSS into well H3 (to provide measurement of any background signal from cellular autofluorescence).
9. Pipette 200 µL of the 75 μM Coumarin dye solution into well H4 (to provide a check on any variation of *t*_0_ during the multiwell plate acquisition, *e.g.*, due to changes in ambient temperature or to fluctuations in laser or timing circuitry.

### 2. openFLIM-HCA Data Acquisition

NOTE: Detailed information can found on the openFLIM-HCA wiki^33^.

1. Start *µManager* and the *OpenFLIM-HCA Plugin*
2. Select the calibration file for the specific combination of delay generator unit and laser repetition rate under "*FLIM control: Delay box calibration file: Calibration Files"* .
3. Set the folder where the data will be saved under "*File: Set Base Folder"*.
4. Check the excitation beam path to ensure that coupling into the delivery optical fiber is stable and that the coupling efficiency is maximized by measuring power transmitted through delivery fiber using a power meter. Fluctuations lower than 5% are acceptable.
5. Check that the GOI triggering is stable by displaying the monitor output signal from the GOI on an oscilloscope triggered by the GOI input trigger signal.
6. Check the imaging of the GOI phosphor onto the CCD sensor by covering the photocathode of the GOI, setting its gain to 750 V and focusing one of the relay lenses such that images of sparse single photon events (due to background light leaking though the GOI cover) recorded on the CCD are as sharp as possible.
7. Check that the sample field of view is uniformly illuminated by imaging a uniform fluorescent sample, such as a drop of the reference dye solution on a coverslip, using a sufficiently low excitation power (< 1 µW) to ensure that the GOI is not saturated.
8. Select the appropriate plate definition file, *i.e.,* select option for 96-well plates ("*File: Load Plate properties"*).
9. Using the *µManager* GUI, ensure that there is no dichroic beam splitter cube in the microscope frame by selecting an empty position.
10. Manually select a gate width of 4 ns at the GOI for the whole experiment.
11. Acquire IRF image data:
  1. Using the *µManager* GUI, ensure that there is no microscope objective in the beam path by selecting an empty position. Manually place a black anodized beam block above the objective turret to prevent any laser light escaping and angle it to minimize any reflection back into the microscope frame.
  2. Using the *µManager* GUI, set the filters in the CSUX (*Excitation, Dichroic, Emission*) such that the fraction of excitation light scattered from the spinning Nipkow disk can be imaged but ensure the intensity is not sufficient to saturate the system for a 200 ms CCD integration time. This is to avoid exposing the GOI photocathode to too much light, which could damage the photocathode.
  3. Use the *Find maxpoint* function over the full range of the delays covered by the programmable fast delay box. Check the output diagram displayed and then adjust the manual course delay box by pushing the forward button so that the full IRF profile is within the scan range of the fast delay box.
  4. Adjust the acquisition parameters (*Neutral density, Exposure time, Frame accumulation*) so the CCD is not saturated in the brightest time-gate and the effective integration time is >200 ms.
  5. Set delay steps of 25 ps over the full delay range ("*FLIM control: Fast delay box: Populate delays"*).
  6. Acquire IRF image by pressing "*Snap FLIM image"*.
  7. Acquire a background image by blocking the laser ("*Light path control: Neutral density: blocked"*), reducing the number of delays ("*FLIM control: Fast delay box: Current delay setting (ps)"*), leave all other properties unaltered and press "*Snap FLIM image"*.
12. Acquire reference dye data:
  1. Select well H4 by using the *µManager* GUI *("XYZ control: Go to well: H4").*
  2. Using the *µManager* GUI, select filters (Excitation 434/17 nm, Dichroic, Emission 482/25 nm) for imaging mTqFP.
  3. Use the *Find maxpoint* function over the full range of the fast delay box and then adjust the coarse delay box so the full decay profile is within the delay range of the fast delay box.
  4. Set the delay to the point of maximum intensity by changing "*FLIM control: Fast delay box: Current delay setting (ps)".* Adjust the acquisition parameters (*Neutral density, Exposure time*) so that the CCD is not saturated.
  5. Set delay steps of 25 ps over the full delay range ("*FLIM control: Fast delay box: Populate delays*).
  6. Acquire reference dye data by pressing "*Snap FLIM image"*.
  7. Acquire a second background CCD camera image by blocking the excitation laser (see 2.11.7)
13. Acquire FLIM data of the multiwell plate sample using the *µManager* acquisition software:
  1. Set which wells should be imaged and the number of FOV to be acquired per well ("*Setup HCA sequenced acquisition: XYZ positions"*).
  2. Manually select an exemplar FOV containing the sample to be imaged. Use this FOV to determine the optimal neutral density filter, gate width at the GOI and integration time of the camera so as to reach 75% of the dynamic range of the CCD camera.
  3. Set autofocus ("*XYZ control: Autofocus")*: Press "*Return focus control only"*, and then manually focus on the cells or structure of interest. Set "*Autofocus search range"* to 2000 µm and press "*Enter"*. Press "*AF now" *to initiate an autofocus procedure. An offset value will show up in the field "*FocusOffset (Autofocus)".*
  4. Enter the offset value obtained in 2.13.3 into "*AF offset"* and repeat the autofocus procedure twice ("*AF now"*) to check that the offset value is now zero, indicating correct functioning of the autofocus system.
  5. Use the "*AutoGate"* function in the *OpenFLIM-HCA* acquisition software to choose a logarithmic sampling of the delay points. Alternatively, these can be selected manually, see reference 36 for more details.
  6. Set "*Accumulate Frames"* to a value that yields desired total data acquisition time. Increasing the number of accumulated frames increases signal to noise ratio of the FLIM data at the expense of also increasing total FLIM data acquisition time.
  7. Run the FLIM acquisition of the multiwell plate by pressing the *"Start HCA sequence"* button.
14. Acquire a further background CCD camera image by blocking the excitation laser (see 2.11.7) with identical acquisition settings as used for the FLIM data acquisition.

### 3. openFLIM-HCA Data Analysis Using *FLIMfit*

1. Check that necessary ancillary files for FLIM data analysis are present (*i.e.*, IRF and reference dye measurement).
2. Load the data from the empty well (H1), the well containing culture medium (H2) and the well containing unlabeled cells in culture medium (H3) into FLIMfit ("*File: Load FLIM data*") to check whether there are any background contributions larger than 1% of the signal from the cells expressing "donor-only", *i.e.,* in wells A1-G3.
3. Prepare a time-varying background (TVB) file by loading the well with culture medium into *FLIMfit ("File: Load FLIM data")*. Load the camera background data acquired in section 2.14 *("Background: Load Background")* and use the *FLIMfit* option "*Tools: Create TVB Intensity Map*" to generate the TVB. See reference 31 for more detail on the TVB.
4. Prepare the spatially varying IRF Shift Map by loading the IRF data acquired in section 2.11 and subtract the CCD camera background acquired in section 2.11.7. Use the *FLIMfit* option "*Tools: Create IRF Shift Map*" to calculate the spatially varying IRF Shift Map. See reference [31] for more detail on the IRF Shift Map.
5. Fit a monoexponential decay to the reference dye data acquired in section 2.12. Load reference dye and the spatially varying IRF Map calculated in section 3.4, set fit parameters (*"Lifetime: No. Exp:1") *with *t*_0_ set as a fitted parameter (*"Lifetime: FIT IRF Shift: Fitted"*). The value of *t*_0_ obtained is then reported in the table shown in the "*Parameters*" tab.
6. Analyze the FLIM data by loading the experimental FLIM data acquired in section 2.13, the spatially varying IRF Map calculated in section 3.4, the camera background acquired in section 2.14, the TVB file prepared in section 3.3 and type in the negative value of *t*_0_ obtained in section 3.5 (*"IRF: IRF shift: t_0_"*). Then fit the experimental data to the desired decay model using *FLIMfit^36^* according to the requirements of the experiment.

## Representative Results

To illustrate the capabilities of the openFLIM-HCA instrumentation, we present three exemplar FRET applications. The first concerns FRET constructs that are adapted from FRET model constructs developed by Stephen Vogel's laboratory^38^. They comprise a series of genetically expressible fluorescent constructs in which a donor fluorescent protein (mTurquoise) is linked to an acceptor fluorophore (Venus) by short controlled lengths of 5, 17 and 32 amino acids (mTq5V, mTq17V, mTq32V). The different linker lengths between donor and acceptor fluorophores result in different FRET efficiencies and therefore different donor lifetimes. To provide a negative control, the Venus in the short 5-amino acid linker construct is replaced by Amber — a non-fluorescent mutation of YFP (mTq5A) that cannot act as a FRET acceptor^38^. We replaced Cerulean in the original pCXV and pC5A vectors by restriction digesting the vectors with NheI and BglII enzymes and ligating mTurquoise fragments digested using the same enzymes from the pmTurquoise-N1 vector. The linker region was unmodified by this substitution.

**Figure 6** presents an exemplar FLIM FRET assay using this model system expressed in Cos cells, in which a multiwell plate is arrayed with 3 columns each of cells transfected with the negative control (columns 1-3), the 5-amino acid linker FRET construct (columns 4-6), the 17-amino acid linker FRET construct (columns 7-9) and the 32 amino acid linker FRET construct (columns 10-12). Row H contains wells for TVB estimation. **Figure 6(a)** shows examples of automatically acquired fluorescence lifetime images of typical fields of view in each well. It is apparent that the negative control (columns 1-3) present the longest lifetimes and that the donor lifetime is lowest for the FRET construct with the shortest linker length (columns 4-6). **Figure 6(b)** shows how the mean lifetime averaged over all the FOVs in each well varies across the multiwell plate. **Figures 6(c) **and** (d) **show exemplar donor fluorescence intensity-merged lifetime maps for a FOV of mTq5A and mTq17V respectively. **Figure 6(e)** shows the donor fluorescence intensity decay profile averaged over all the cells imaged in well A1, together with the fit to a monoexponential decay model and the IRF (which is dominated by the GOI gate width). **Figure 6(f)** presents the average fluorescence lifetime for each column of the multiwell plate obtained from a pixel-wise monoexponential fit. A table of the mean lifetime and standard deviation (STD) can be found in the **Table 1** below. The data acquisition for the images shown in **Figure 6 **took approximately 160 min to acquire. The analysis took 92 s on a 2.6 GHz computer with 10 cores and 64 GB of RAM.

The second exemplar assay demonstrates the application of FLIM FRET to read out the oligomerization of HIV-1 Gag during the assembly of HIV-1 virions as a means to test inhibitors of this process^25,42^. Expressing HIV-1 Gag in appropriate host cells provides a model system for this stage of the HIV-1 lifecycle since it leads to the formation of viral-like particles (VLPs) that are similar to immature HIV-1 virions. For this assay, we expressed HIV-1 Gag protein fused with CFP in HeLa cells and compared the action of different inhibitors via their impact on the FRET signal that resulted from the Gag oligomerization. Details of the inhibitors used are provided in reference 42.

The details of the sample preparation and experimental measurements are described comprehensively in reference 25, where we demonstrated that we could use FLIM to read out both heteroFRET between aggregating Gag proteins labeled stochastically with CFP and with YFP, and also homoFRET in cells expressing only Gag labeled with CFP. Although homoFRET does not usually present a change in donor lifetime, it does for the special case of CFP where the fluorophore exists in two isomers with different mean fluorescence lifetimes^40,41^. The CFP homo-FRET readout has the advantages of simplified sample preparation compared to CFP/YFP hetero-FRET and is more spectral efficient, which could be important for multiplexed FRET readouts.

Our previous work applied FLIM FRET to assay the Gag oligomerization in the presence of an N-myristoyltransferase (NMT) inhibitor^42^. This inhibitor disrupts the endogenous enzymes responsible for the addition of myristic acid to Gag, which enables Gag proteins to bind to the plasma membrane and is a prerequisite^43^ in the formation of HIV virions or VLPs. We showed that both heteroFRET and homoFRET readouts could achieve Z' of >0.6 in dose response studies with an NMT inhibitor. Z' is a parameter used in the pharmaceutical industry to indicate the quality of an assay and represents the differences in the mean values of the positive and negative controls and their relative spreads to generate a single value metric of assay quality — with *Z'* > 0.5 being considered "excellent" for a pharmaceutical assay^44^. We note that this excellent performance was achieved with samples for which the change in mean donor lifetime was of the order of 300 ps — demonstrating the statistical power of analyzing FLIM data for large numbers of cells, which enables useful readouts to be realized using much smaller changes in fluorescence lifetime than is possible with the typical numbers of cells imaged in manual microscopy experiments.

Here, we present a multiwell plate FLIM FRET data set comparing 4 inhibitor compounds for HIV Gag oligomerization (designated ICL13, ICL14, ICL15 & ICL16). For this experiment, we utilized the homoFRET readout with HeLa cells transiently transfected with the Gag-CFP plasmid. A total of nine different doses of each inhibitor were applied following the standard protocol outlined in reference 32, allowing two repeat wells per condition. As a positive control, column 1 presents cells expressing myr-Gag, a mutated Gag protein lacking the myristic acid moiety that cannot form VLPs and so simulates total inhibition. A negative control was provided by dosing cells expressing Gag-CFP only with just the vehicle used to deliver the inhibitors in the other columns (column 11). A total of eight FOV were acquired per well.

Data was fitted with a single exponential decay model on a pixel-by-pixel basis and the fluorescence lifetime plate map showing the average lifetime per well (over all pixels above the intensity threshold across the eight fields of view) is shown below in **Figure 7(a)**. **Figure 7(b)** shows the corresponding plot of fluorescence lifetime as a function of inhibitor concentration. Three of the inhibitors show an inhibitory effect (ICL13, ICL15 & ICL16) when incubated with cells expressing Gag-CFP. One inhibitor (ICL14) shows no effect at any dose tested. The Z' calculated for this plate was 0.51. The values obtained for the positive and negative controls are shown on **Figure 7(b)** as the points in black at 100 µM and 0.1 nM.

The dose response curves for ICL13, ICL15 and ICL16 shown in **Figure 7(b)** were then fitted to the 4 parameter logistic nonlinear regression model with the Hill coefficient fixed to 1^45^ with the maximum and minimum lifetimes fixed to the values obtained from the positive (column 1) and negative (column 11) controls respectively. The returned IC_50_ values for the three curves were then: 38 nM, 24 nM and 116 nM for ICL13, ICL15 and ICL16 respectively. For comparison with IC_50_ values obtained through intracellular FLIM FRET measurements, we determined IC_50_ for inhibition of enzymatic activity of recombinant human NMT1 in our previously reported biochemical enzyme assay^42^. The three compounds found to be active by FLIM FRET are also the most active in this assay (IC_50_ values of 17 nM, 51 nM, and 39 nM for ICL13, ICL15 and ICL16, respectively), with ICL14, which showed no significant response in the FLIM FRET assay, being *ca.* 100-fold less active against NMT1 (IC_50_ of 1700 nM). For the active compounds, the IC_50_ values obtained through these independent assay modalities are remarkably similar, with minor variations likely attributable to differences in uptake or metabolism of the compound in cells.

This small study highlights the ability of FLIM HCA to discriminate the potency of different compounds which act on the same target of interest. We believe it is the first demonstration of a screen using automated FLIM in a high content context to generate multiple dose response curves and illustrates the potential for FLIM to be applied to screen for and/or characterize useful therapeutic compounds.

The third exemplar FLIM FRET experiment concerns a genetically expressed FRET biosensor for Exchange protein activated by cyclic adenosine monophosphate (cAMP (EPAC)), which is a Rap-1 guanine exchange factor that binds specifically to cAMP. This EPAC FRET biosensor (EPAC-S^H188^) utilizes mTurquoise2 fluorescent protein (mTq2FP) as the donor fluorophore and incorporates two Venus-FP to implement a composite acceptor^46^. cAMP is a ubiquitous second messenger and is involved in a plethora of intracellular processes throughout many different organisms. It is synthesized by adenylate cyclase from the conversion of ATP at the cell membrane. Here we demonstrate our ability to read out and quantify its activity in live HEK293T cells following the addition of forskolin, an adenylate cyclase activator that upregulates intracellular production of cAMP and therefore increases its cytoplasmic concentration. This EPAC sensor undergoes FRET in its inactivated (unbound) state and its donor lifetime increases with cAMP concentration as the cAMP leads to the opening of the biosensor. The mono-exponential decay profile of mTq2FP makes this biosensor better suited for quantitative lifetime analysis than CFP-based biosensors ^45^. **Figure 8** shows an example of a time course recorded using the automated multiwell plate FLIM microscope where we have plotted the change in mean lifetime and the change in the FRETing donor population fraction over time following the addition of 100 µM forskolin. For simplicity, we assume that the total signal can be described by a mixture of monoexponential FRETing and non-FRETing donors and the population fraction of the activated EPAC FRET biosensor was obtained by fitting a double exponential decay profile across the time series.

For the data acquisition of cAMP (EPAC) five gate delays, with a gate width of 4,000 ps, were chosen with the delay time set to 1,300 ps, 4,300 ps (peak intensity), 4,919 ps, 6,656 ps, 8,035 ps, 1,0391 ps and 16,000 ps. The camera integration time for each delay was 250 ms. At one well, we acquired a FLIM time-course with images acquired every 10 s over 10 min. The data points acquired at times 120 s and 130 s were removed because the addition of forskolin caused a small shift in the focal position of the sample and therefore of the fluorescence intensity recorded during the FLIM acquisitions at these time points.

For the data analysis the images were loaded into *FLIMfit *and segmented per cell. The fluorescence lifetime data was fitted to a double exponential decay model using an IRF and a fixed *t*_0_ value obtained from a reference dye (75 µM Coumarin 6). The donor fluorescence mean lifetime averaged over all cells is shown in **Figure 8(a)**. **Figure 8(b)** shows the change in FRETing population fraction over time calculated by fitting the same FLIM data to the same double-exponential decay model


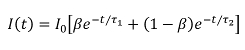


where *β* is the FRETing donor fraction. We assume a mixture of FRETing and non-FRETing elements for the time points prior to addition of forskolin. To obtain these lifetimes, a double exponential fit was applied to the first three time points giving lifetimes of τ_1_ = 3,964 ± 21 ps for the non-FRETing donor, and τ_2_ = 3,022 ± 27 ps for the FRETing donor. These were fixed for the subsequent fit of the whole data set to obtain the *β* values at each time point.

In summary, we have presented exemplar FLIM HCA results for three experimental samples. The first was a 96 well plate arrayed with Cos cells expressing FRET constructs comprising a mTqFP donor linked to either a Venus FP acceptor or a non-fluorescent Amber construct by varying lengths of peptide linker. The change in FRET efficiency and therefore donor lifetime with linker length is clearly apparent and the standard deviation of the mean lifetime for each construct was less than 36 ps. The second exemplar assay was a "screen" of four potential inhibitors for myristoylation of the HIV Gag protein for which the % inhibition was calculated from the variation of mean donor fluorescence lifetime with inhibitor concentration measured in Hela cells expressing Gag protein labeled with CFP. This readout is based on homoFRET, which would not usually present a change in donor lifetime but does for CFP because this exists in multiple isomers with different fluorescent lifetimes. This can be useful in terms of spectral efficiency, *e.g.,* for multiplexing different FRET readouts^25^. The third exemplar assay was a time course monitoring live HEK293T cells expressing the cAMP FRET biosensor, EPAC. This showed the expected response following treatment with forskolin and the application of *FLIMfit* using a double exponential decay model with the number of detected photons being commensurate with live cell imaging — as we have previously shown with the LIBRA FRET biosensor for IP3^47^.


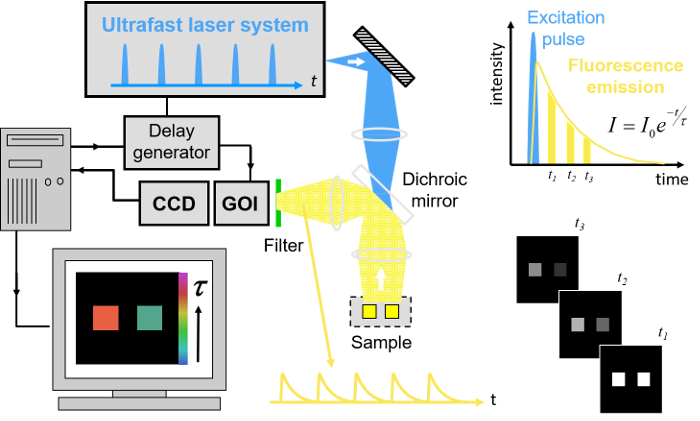
**Figure 1. Schematic of wide-field time-gated FLIM using a gated optical image intensifier (GOI). **Left hand side, shows a schematic of the experimental system. Top right, schematic of excitation pulse, position of time-gated images and monoexponential fit to data. Bottom right, cartoon showing the fluorescence decay from the two objects shown in the experimental system. Please click here to view a larger version of this figure.


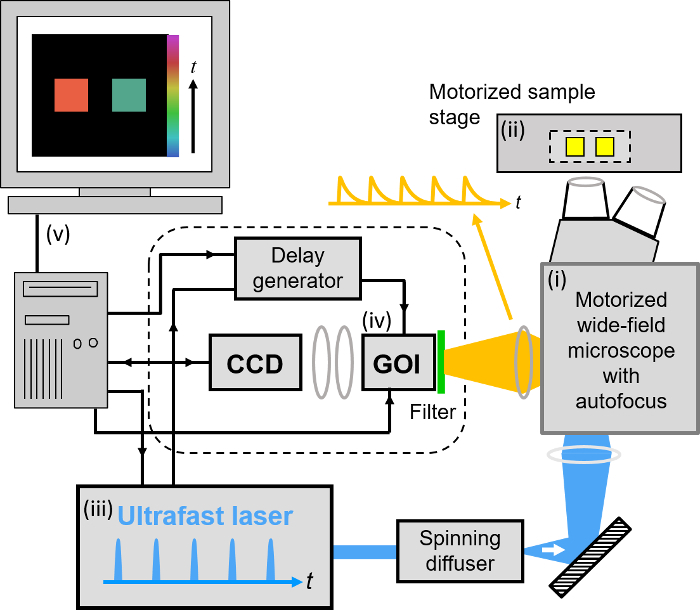
**Figure 2. Schematic of wide-field openFLIM-HCA using a gated optical image intensifier (GOI). **See main text for more detailed description of the system components. Please click here to view a larger version of this figure.


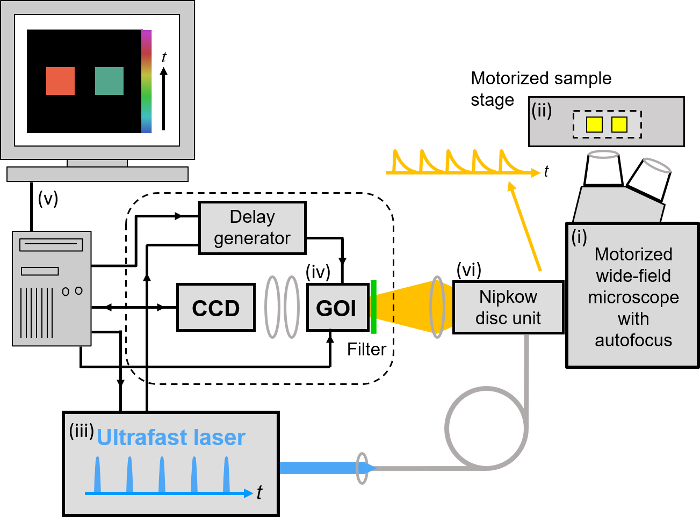
**Figure 3. Schematic of optically sectioned openFLIM-HCA using a gated optical image intensifier (GOI) with a Nipkow disc scanner unit. **See main text for more detailed description of the system components. Please click here to view a larger version of this figure.


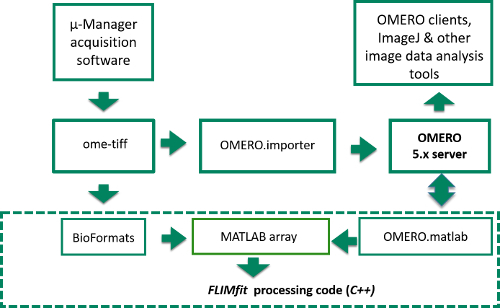
**Figure 4. Schematic of image data flow from Manager acquisition program to OMERO server or computer disk and into FLIMfit for analysis. **Data flow starts in top left corner where raw image data is acquired in μ-Manager acquisition software. The items inside the dashed box represent the stages of the FLIM data analysis, while those outside correspond to the data acquisition and storage. Please click here to view a larger version of this figure.


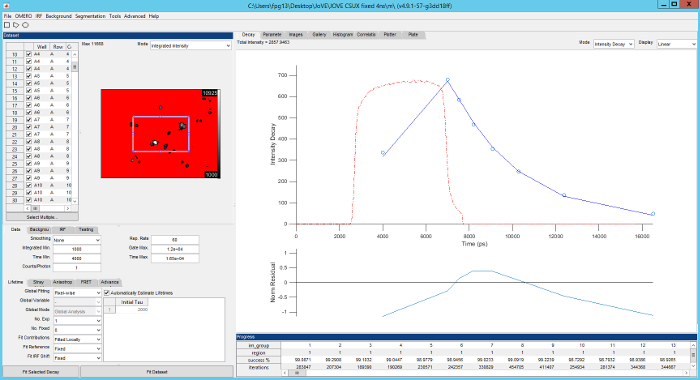
**Figure 5. Screenshot of FLIMfit user interface. **Top left, list of fields of view currently loaded and fluorescence intensity image of the currently selected field of view. Bottom left, selection of fitting model and fitting conditions. Top right, fluorescence decay for current region of interest (blue circles), fit to data (red line), IRF (red dashed line) with fit residuals below (blue line). Please click here to view a larger version of this figure.


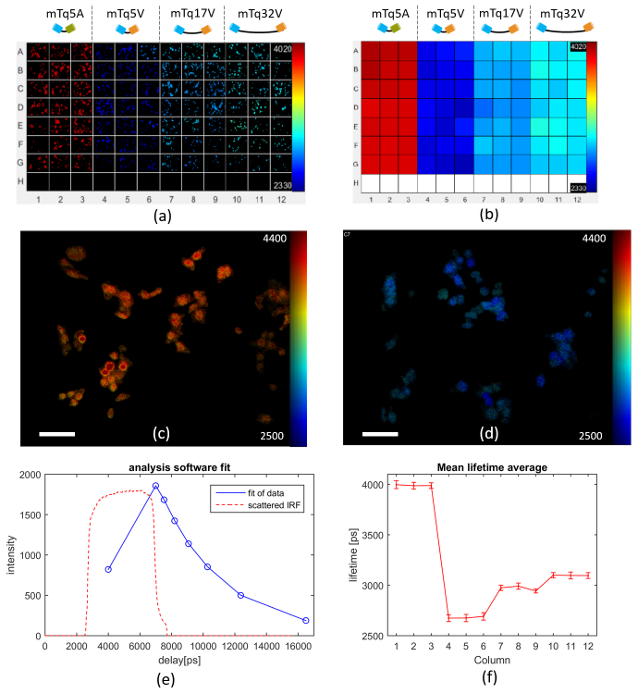
**Figure 6. Multiwell plate FLIM of FRET model constructs mTq5V, mTq17V, mTq32V expressed in Cos cells. **(**a**) exemplar fluorescence lifetime images of FOV in each well for different FRET constructs expressed in Cos cells as discussed in text. (**b**) heat map of mean Venus fluorescence lifetimes averaged over all cells in each well. (**c**) intensity image of mTurquoise Amber overlaid with lifetime map (scale bar 20 µm). (**d**) intensity image of mTurquoise Venus (17 amino acid) overlaid with lifetime map (scale bar 20 µm). (**e**) measured intensity decay profile (blue circles) of mTq5A construct (negative control) fluorescence averaged over all the cells imaged in well A1, together with the fit of the data to a monoexponential decay (blue solid line) and IRF (dashed orange line). (**f**) graph of mean lifetime averaged over each column across the multiwell plate, with error bars showing the standard deviation in fluorescence lifetime between fields of view. For (a-d) lifetime values are represented using a false color scale with the indicated limits in picoseconds. Please click here to view a larger version of this figure.


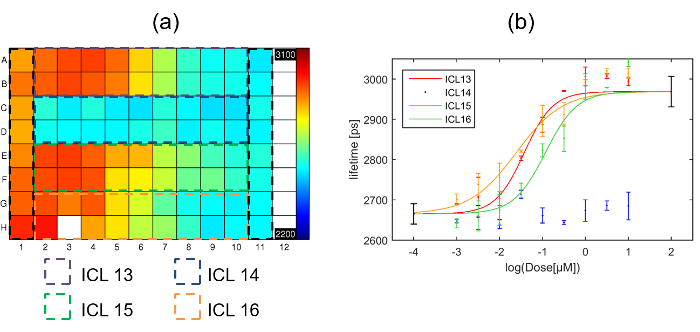
**Figure 7. Exemplar multiwell plate FLIM screen of Gag inhibitors using HeLa cells expressing Gag-CFP. **(**a**) Heat map of mean donor fluorescence lifetime per well for multiwell plate arrayed with column 1 presenting cells transfected with myr-Gag-CFP as a positive control for inhibition, column 11 presenting cells transfected with Gag-CFP exposed to vehicle only, and the dashed colored squares indicating the wells that were dosed with one of the four different inhibitors with concentrations ranging from high dose column 2 to low dose column 10. The false color scale represents the mean fluorescence lifetime ranging from 2,200 ps to 3,100 ps. (**b**) plots fluorescence lifetime for each inhibitor with error bars showing the standard deviation between repeat wells. Points in black at 2 and -4 on the horizontal axis are the positive and negative control points respectively obtained from columns 1 and 11 respectively. The solid lines indicate a fit to the dose response curve data for the different inhibitors. Please click here to view a larger version of this figure.


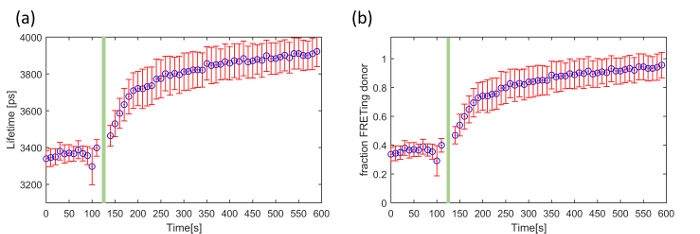
**Figure 8. Exemplar use of FLIM to read out the EPAC FRET biosensor in a time-lapse measurement using HEK293T cells. **Change in (**a**) mean fluorescence lifetime and (**b**) fraction of FRETing donor as a function of time following addition of forskolin indicated by the green bar. The error bars show the standard error per cell. Please click here to view a larger version of this figure.

**Table:** 

	**per Well**	**STD**	**ERR**			**per Well**	**STD**	**ERR**
mT5A	4008	32	12		mT17V	3004	26	10
mT5V	2717	35	13		mT32V	3133	30	11

**Table 1. Mean lifetime and standard deviation (STD) of the FRET constructs.**

## Discussion

We have described an automated multiwell plate microscope designed for HCA using time-gated FLIM. This instrument is controlled using open source software written in *µManager* with the option of saving the data to an OMERO server and the FLIM data analysis being undertaken using *FLIMfit^36^, *an open source program written in MatLab and available as an OMERO client^32^ The *µManager* instrument control software, *openFLIM-HCA*, is available online^33^ with a list of the system components^48^ to enable academic researchers to construct their own FLIM HCA instruments.

In order to acquire robust FLIM data, it is necessary to optimize the GOI and CCD acquisition settings and to take account of any variations in *t*_0_. As described in 3.8.2, the GOI gain setting and CCD camera integration time should be set to reach ~75% of the CCD dynamic range. Using higher GOI voltages and multiple CCD frame accumulations at each time gate delay increases the signal to noise ratio^49^ but this increases the total FLIM acquisition time (since fewer fluorescence photons are acquired per frame before the CCD camera saturates and so the number of frame accumulations should be increased) and so this trade-off should be decided for each experiment. The calibration of the delay generator depends on the laser repetition rate and it is therefore necessary to ensure that the correct calibration file for the repetition rate used is loaded into the acquisition software. The procedure for calibrating the delay box can be found on the openFLIM-HCA wiki^33^.

If the cellular autofluorescence is low compared to the fluorescence signal, we use the signal from a well containing just culture medium to provide the time-varying background (TVB). To minimize background fluorescence from the cell culture medium, we avoid media containing phenol red. If the cellular autofluorescence is significant, then the TVB may be derived from measurements of the unlabeled cells. However, in this case care must be taken, as this assumes that the autofluorescence background is the same for every pixel in the image. This assumption will not be valid if the autofluorescence varies significantly across a cell or if the excitation beam or detection sensitivity is not uniform over the field of view.

The acquisition of an IRF image using scattered excitation light pulses provides the temporal IRF profile that is convolved with the data model in the fitting process and also provides a map of the spatial variation of the IRF across the field of view. The IRF can be measured by imaging a scattering sample such as a colloidal suspension although, in our instrument, using excitation light scattered from the Nipkow disk provides a cleaner measurement of the IRF. Accurate determination of the timing of the IRF is important because errors in the time delay between the excitation and time-gating can significantly impact the fitting process, particularly when fitting to complex exponential decay models. The IRF acquired using scattered excitation light is not exactly what is needed for the fitting process because it is recorded at the excitation wavelength rather than at the fluorescence emission wavelength, which can affect its timing, although the spatial variation of the IRF should be the same at both wavelengths. In addition, the scattered IRF may be globally shifted in time relative to the true IRF due to a differing optical path length from the scattering object, as is the case for an IRF acquired from the Nipkow disk in optically sectioned FLIM. This correction,* t*_0_, which is the required global temporal shift that should be applied to the acquired scattered excitation light IRF, is calculated from the monoexponential reference dye data as indicated in Protocol step 4.4. The following dyes can be used for different excitation wavelengths: a 75 μM Coumarin 153 solution in methanol (τ ~ 4.3 ns^50^) can be used for excitation in the range 295-442 nm; a 75 μM Coumarin 6 solution in ethanol (τ ~ 2.43 ns^37^) can be used for excitation in the range 430-500 nm; and a 75 μM Rhodamine B solution in water (τ ~ 1.7 ns^37^) can be used for excitation in the range 488-575 nm. We note that, although it is possible in *FLIMfit* to use a different IRF for each pixel of the system, usually it is reasonable to make the assumption that the spatially varying IRF has the same temporal profile for every pixel in the image but presents a range of spatially varying temporal offsets relative to the global *t_0_*. We describe this as the IRF shift map, which is determined from the scattered light IRF. Together, the IRF profile, *t*_0_ and the IRF shift map are used to ensure that each pixel in the FOV is fitted with an appropriate IRF. Importantly, *t*_0_ can vary slowly over time so it should be measured before any FLIM data acquisition.

The user should decide how to sample the fluorescence decay profiles and is required to set the width of the time-gates and their relative delays after excitation. In general, it is desirable to use broad gate widths to maximize the detected signal and typically we use gate widths set to 4 ns for FLIM of cells labeled with fluorescence proteins. The number of time-gate delays should be sufficient to sample the fluorescent decay profile (including one gate set to measure the signal just before the excitation pulse) given the complexity of fitting model that will be used in the analysis. The optimum value can depend on the lifetimes and relative amplitudes of the decay components. In general, 4 gates are sufficient for fitting monoexponential decays while 7 or more gates may be used for more complex decay models.

We note that FLIM can be implemented using a range of time-domain or frequency domain techniques and automated FLIM HCA of multiwell plate arrays was first implemented in a (non-sectioning) wide-field microscope using frequency domain lifetime readouts of FRET^51^. The first automated optical sectioning FLIM multiwell plate reader for HCA utilizing wide-field time-gated imaging^28^ was then reported. Subsequently, wide-field (non-sectioning) frequency domain FLIM HCA was applied to screen for post translational modifications (tyrosine phosphorylation) across a gene library using FRET^52^ and time-gated FLIM HCA has been applied to FRET assays of HIV-1 Gag oligomerization^53^ and of SUMOylation of FOXM1^54^ and of Raichu RhoA and Rac1 biosensors^33^. It is also possible to use TCSPC for multiwell plate FLIM^26^ but to date it has proved challenging to match the throughput of techniques exploiting wide-field detection. One emerging approach that could address this issue is the use of arrays of single photon counting detectors such as SPAD arrays^55^.

We note that any readouts of FRET using fluorescent proteins should be treated with caution and that the absolute values of parameters obtained from *FLIMfit* or any other FLIM analysis software will depend on the assumptions associated with the fitting model. For example, where the FRET donor has a significant second decay component, the use of a biexponential model to fit the FRET FLIM data can result in the contribution of the faster decay component, typically associated with the FRETing population appearing higher than it should. It is therefore desirable to use donors with a mono-exponential lifetime such as mTq2FP. Even then, recent studies have shown that FRET analysis can still be affected by dark states of acceptor fluorescence proteins or by heterogeneity of the orientation factor κ^2^ due to a distribution of fluorophore conformations with a variety of chromophore angles and distances^56^. Nevertheless, we and others have shown that fluorescence lifetime readouts of FRET do produce useful results that correlate with biochemical measurements and can be used to screen for protein interactions or to follow dynamics of biosensors. Thus this openFLIM HCA platform can be applied to a wide range of fluorescence lifetime-based assays utilizing FRET or cellular autofluorescence^8^, as well as providing quantitative readouts of dye-based probes^25^.

## Disclosures

The authors have nothing to disclose. The funders had no role in study design, data collection and analysis, decision to publish, or preparation of the manuscript.
